# Interplay between the Gut Microbiome and Metabolism in Ulcerative Colitis Mice Treated with the Dietary Ingredient Phloretin

**DOI:** 10.4014/jmb.2104.04038

**Published:** 2021-08-09

**Authors:** Jie Ren, Puze Li, Dong Yan, Min Li, Jinsong Qi, Mingyong Wang, Genshen Zhong, Minna Wu

**Affiliations:** 1 School of Basic Medical Sciences, Xinxiang Medical University, Xinxiang 453003, Henan, P.R. China; 2 Department of Interventation, The First Affiliated Hospital of Xinxiang Medical University, Xinxiang 453003, Henan, P.R. China; 3 Henan Key Laboratory of Immunology and Targeted Therapy, Henan Collaborative Innovation Center of Molecular Diagnosis and Laboratory Medicine, Xinxiang Medical University, Xinxiang 453003, Henan, P.R. China

**Keywords:** Phloretin, ulcerative colitis, faecal metabolite, gut microbiota

## Abstract

A growing number of healthy dietary ingredients in fruits and vegetables have been shown to exhibit diverse biological activities. Phloretin, a dihydrochalcone flavonoid that is abundant in apples and pears, has anti-inflammatory effects on ulcerative colitis (UC) mice. The gut microbiota and metabolism are closely related to each other due to the existence of the food-gut axis in the human colon. To investigate the interplay of faecal metabolites and the microbiota in UC mice after phloretin treatment, phloretin (60 mg/kg) was administered by gavage to ameliorate dextran sulfate sodium (DSS)-induced UC in mice. Gut microbes and faecal metabolite profiles were detected by high-throughput sequencing and liquid chromatography mass spectrometry (LC-MS) analysis, respectively. The correlations between gut microbes and their metabolites were evaluated by Spearman correlation coefficients. The results indicated that phloretin reshaped the disturbed faecal metabolite profile in UC mice and improved the metabolic pathways by balancing the composition of faecal metabolites such as norepinephrine, mesalazine, tyrosine, 5-acetyl-2,4-dimethyloxazole, and 6-acetyl-2,3-dihydro-2-(hydroxymethyl)-4(1H)-pyridinone. Correlation analysis identified the relations between the gut microbes and their metabolites. *Proteus* was negatively related to many faecal metabolites, such as norepinephrine, L-tyrosine, laccarin, dopamine glucuronide, and 5-acetyl-2,4-dimethyloxazole. The abundance of unidentified *Bacteriodales_S24-7*_group was positively related to ecgonine, 15-KETE and 6-acetyl-2,3-dihydro-2-(hydroxymethyl)-4(1H)-pyridinone. The abundance of *Christensenellaceae*_R-7_group was negatively related to the levels of 15-KETE and netilmicin. *Stenotrophomonas* and 15-KETE were negatively related, while *Intestinimonas* and alanyl-serine were positively related. In conclusion, phloretin treatment had positive impacts on faecal metabolites in UC mice, and the changes in faecal metabolites were closely related to the gut microbiota.

## Introduction

Many of the ingredients in dietary plants are beneficial for human health, and the effects of these ingredients are noteworthy. Researchers have focused on the regulatory effects of these ingredients and their use in disease prevention and treatment. Phloretin, a dihydrochalcone flavonoid extracted from apples, pears, litchis and other juicy fruits, has shown diverse biological activities, such as antioxidant, glucose transporters-regulating, and antibacterial activities [1, 2].

Ulcerative colitis (UC), an inflammatory bowel disease (IBD), is limited to the mucosa and submucosa of the colon and rectum [3]. UC patients usually exhibit weight loss, abdominal pain and bloody diarrhoea. However, the aetiology of UC remains unclear, which has resulted in a poor treatment strategy. It has been reported that the gut microbiota, gene susceptibility, dietary habits and hygiene are all correlated with the development of UC [4]. No specific diet has been demonstrated to directly relate to the occurrence of UC; however, dietary intervention, such as consumption of a fasting-mimicking diet, can reduce colon inflammation [5], and consumption of a fiber-deprived diet, a high-salt diet and a high-sugar diet can increase susceptibility to colon inflammation [6-8]. Further investigation has shown that the fasting-mimicking diet, the high-salt diet and the fiber-deprived diet can influence the structure and composition of the gut microbiota. The gut is the largest habitat for bacteria, and there are approximately 4 × 10^13^ bacteria in the gut [9], and studies have shown that the gut microbiota participates in immune system formation, intestinal barrier integrity, and tryptophan metabolism regulation [10-13]. Some bacteria, such as butyrate-producing bacteria ferment undigested complex carbohydrates to produce short-chain fatty acids (SCFAs), which can regulate host physiology and immune functions [12].

Due to the existence of the human food-gut axis and the importance of the gut microbiota in metabolizing small molecules, the gut microbiota and faecal metabolites are thought to be closely related [14]. Many studies have focused on the effects of gut microbial taxa on health and disease status, but changes in metabolism, especially changes in metabolites, also deserve to be investigated. With the rapid development of metabolic sequencing technology, liquid chromatography-mass spectrometry (LC-MS) and gas chromatography-mass spectrometry (GC-MS) have been broadly used for targeted or nontargeted metabolomics analyses of mice and patients, which have helped researchers to comprehensively understand the underlying mechanisms of diseases. The metabolic activity of the gut microbiota is essential for maintenance of host homoeostasis and health [15]. Shifts in the gut microbiota can cause metabolic profile changes, and the changes in metabolites subsequently affect the immune response and cell proliferation, eventually resulting in health changes or disease [16, 17]. For instance, dysbiosis of the gut microbiota is associated with metabolic diseases, such as type 2 diabetes, obesity and metabolic syndrome [18-20]. Franzosa *et al*. reported changes in the faecal metabolites of UC and Crohn’s disease (CD) patients with disturbed gut microbiota [21], which enhanced the understanding of the microbiota-metabolism relationship in IBD patients. Yu *et al*. also demonstrated variations in the gut microbiota and faecal metabolites in depressed mice [22]. Therefore, it is vital to assess gut microbiota-related metabolic changes, and further evaluate the mechanisms for the treatment of disease.

Our previous study demonstrated that phloretin exerts an obvious ameliorative effect on UC mice by regulating the gut microbiota [23]. Alsanea *et al*. demonstrated that phloretin can significantly regulate glucose and lipid metabolism in obese mice [24]. However, there have been no studies about the effects of phloretin on faecal metabolites. In this study, we aimed to investigate the changes in faecal metabolites after phloretin treatment and further analyse the correlations between the gut microbiota and faecal metabolites in phloretin-treated UC mice.

## Materials and Methods

### Animals and Reagents

Eight-week-old SPF male C57BL/6 mice weighing 21-22 g were obtained from Beijing Vital River Laboratory Animal Technology Co., Ltd. All mice were kept in individually ventilated caging (IVC) systems at 23°C ± 2°C under a humidity of 55% ± 5% and a 12-h light/dark cycle. The mice were allowed to eat chow food and drink water ad libitum. The study was approved by the Ethics Committee of Xinxiang Medical University (XYLL-2018-B001), Xinxiang City, China. And the animal experiments were carried out in accordance with the National Institutes of Health Guide for the Care and Use of Laboratory Animals. Dextran sulfate sodium (DSS, molecular weight of 36-50 kDa) was purchased from MP Biochemicals (USA). Phloretin (molecular weight of 274.27 Da) was purchased from Dalian Meilun Biological Technology Co., Ltd. (China).

### Experimental Design

After one week of adaptive feeding, the mice were randomly divided into three groups (n =6 for each group): the healthy control group (Con), the ulcerative colitis group (DSS) and the 60 mg/kg/d phloretin-treated UC group (DSSPh). The mice in the DSS group were free to drink sterile water from the first to the third day. Ulcerative colitis was induced from the 4^th^ day to the 10^th^ day with 3% DSS in drinking water as previously described [25]. Then, the mice were free to drink sterile water from the 11^th^ day to the 17^th^ day. Phloretin (60 mg/kg/d) was administered by gavage from the 1^st^ day to the 17^th^ day as described in our previous study [23]. The mice in the Con group and the DSS group were given equal volumes of sterile water intragastrically. The mice were sacrificed by cervical dislocation under ether anaesthesia on the 17^th^ day, and fresh faeces were collected.

### Disease Activity Index (DAI)

The body weight of each mouse was recorded every day. Stool consistency and gross blood were measured every day during the DSS administration and recovery periods. The DAI was calculated as the average score of body weight loss, stool consistency and gross blood, as described previously [26].

### Real-Time Quantitative Polymerase Chain Reaction (RT-qPCR)

Colon tissues were collected at the end of the experiment. RNA in colon tissues was extracted using TRIzol reagent (Tiangen, China). Reverse transcription was performed using PrimeScript RT master mix (Takara, China). Quantitative PCR was conducted in an ABI StepOne Plus Real-Time system with TB Green PCR Core Reagent (Takara). The mRNA expression of cytokines was calculated by the ^ΔΔ^Ct method. The primer sequences are shown in Table 1.

### 16S rDNA Gene High-Throughput Sequencing

On the 17^th^ day, 60 mg of fresh faeces was collected. Total DNA was extracted according to the manufacturer’s protocol (Biomiga, China). Agarose gel electrophoresis was used to detect the quality of the DNA, and a NanoDrop 2000 ultraviolet spectrophotometer was used to measure the purity and concentration of the DNA. The V3-V4 region gene of the bacterial genome was amplified using the universal primers 338F (5′-ACTCCTACGGGAGGCAGC-3′) and 806R (5′-GGACTACHVGGGTWTCTAAT-3′) [27]. The PCR products were purified, and the concentrations were adjusted. Then, sequencing was performed on an Illumina MiSeq PE300 system (MajorBio Co., Ltd., China). The raw gut microbiota data have been uploaded to the NCBI Sequence Read Archive (SRA). The accession number is SRP280243.

### Untargeted Faecal Metabolomics Profiling

**Sample preparation**. On the 17^th^ day, 100 mg of fresh faeces was collected into an Eppendorf tube, and then placed in liquid nitrogen for rapid freezing. The metabolites were extracted following the manufacturer’s instructions (Biotree Biotech Co., Ltd, China). One millilitre of extract solution (acetonitrile: methanol: water = 2:2:1, with isotopicallylabelled internal standard mixture) was added to an Eppendorf tube. After vortexing for 30 s, the samples were ground at 35 Hz for 4 min, and sonicated for 5 min on ice. The grinding and sonication treatments were repeated for 3 times. After that, the samples were incubated at -40°C for 1 h and then centrifuged at 12,000 rpm and 4°C for 15 min. The supernatant was collected for LC-MS analysis. The quality control (QC) sample was prepared by mixing equal volumes of supernatant from all test samples.

**LC-MS metabolomics processing**. LC-MS analysis was performed on a UHPLC-QE-MS system (Vanquish, Thermo Fisher Scientific), equipped with a UPLC BEH Amide column (2.1 mm × 100 mm, 1.7 μm). The mobile phase consisted of A (containing 25 mmol/l ammonium acetate and 25 mmol/l ammonia hydroxides in water, pH = 9.75) and B (acetonitrile). The column temperature was maintained at 25°C, and the elution gradient was: 95% B, 0~0.5 min; 65%~95% B, 0.5~7.0 min; 40%~65% B, 7.0~8.0 min; 40% B, 8.0~9.0 min; 40%~95% B, 9.0~9.1 min; and 95% B, 9.1~12.0 min. The injection volume was 2 μl, and the auto-sampler temperature was 4°C.

Under the control of the acquisition software (Xcalibur, Thermo), a Q Exactive HFX mass spectrometer (Orbitrap MS, Thermo) was used to collect the mass spectrometric data in information-dependent acquisition (IDA) mode. The detailed parameters are as follows: sheath gas flow rate, 50 Arb; aux gas flow rate and capillary temperature, 10 Arb and 320 °C; full MS resolution, 60000; MS/MS resolution, 7500; collision energy, 10/30/60 in NCE mode; and spray voltage, 3.5 kV positive.

**Data processing and analysis**. Raw peak detection, extraction, alignment and integration were performed with the R software package. Then, an in-house MS2 database (Biotree DB) was applied for metabolite annotation. The cut-off for annotation was set at 0.3.

### Statistical Analysis

The data were analysed with GraphPad Prism 5.0 software using one-way analysis of variance (ANOVA) followed by Tukey’s honestly significant difference (HSD) multiple comparison post hoc test. The data are expressed as the mean ± standard error of the mean (SEM). *p* < 0.05 was considered to indicate statistical significance.

## Results

### Protective Effect of Phloretin on UC Mice

As shown in Fig. S1, the DSS group mice had lower body weights and higher DAIs than the Con group mice (*p* < 0.001), while phloretin administration markedly increased the body weights and decreased the DAIs of the DSSPh group mice (*p* < 0.05). DSS group mice had higher mRNA levels of pro-inflammatory cytokines, including TNF-α, IL-6 and IL-1β, and lower mRNA levels of IL-10 than Con and DSSPh group mice; all of these findings are consistent with those of our previous study[23].

### Structure of the Faecal Microbiota

**Fig. 1:**
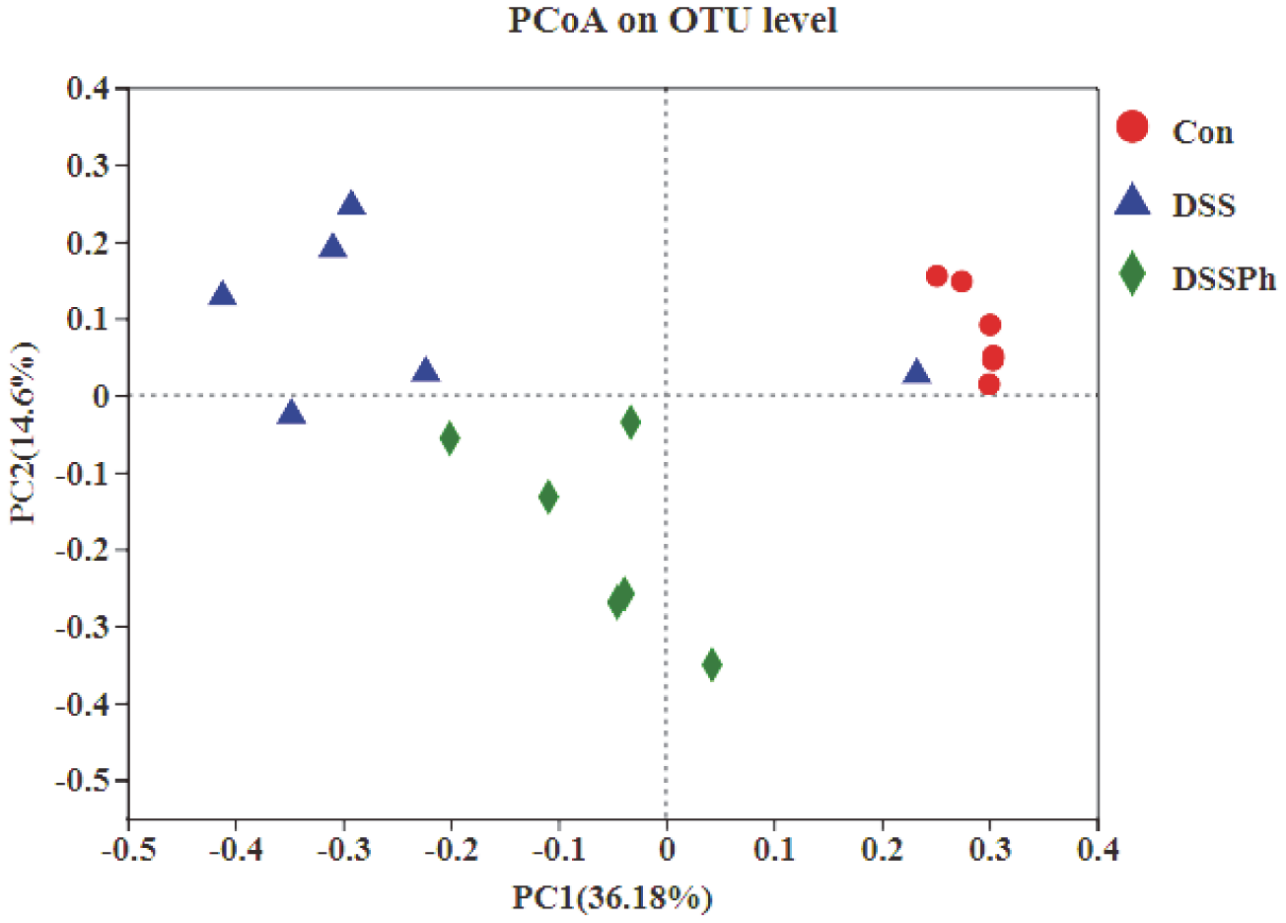
PCoA analysis of the gut microbiota. Con, healthy control group; DSS: DSS-induced UC group; DSSPh, phloretin-treated UC group (60 mg/kg/d). *n* = 6 for each group.

The results of PCoA based on the Bray-Curtis distance are shown in Fig. 1. The distance from the DSSPh group to the Con group was shorter than that from the DSS group to the Con group, which indicated that the structure of DSSPh mice was more similar to that of Con group mice than to that of DSS group mice. Consistent with the findings of our previous study [23], phloretin treatment improved the gut microbial structure of UC mice.

The influences of phloretin on gut microbial composition in UC mice have been shown in detail in our previous study, so the results of this research described below mainly show the effects of phloretin on mouse faecal metabolites.

### Global Views of Faecal Metabolites Profiles

**Fig. 2:**
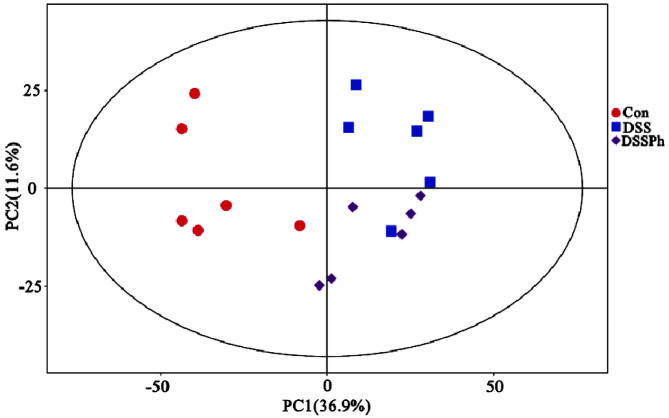
PCA analysis of faecal metabolites. Con, healthy control group; DSS: DSS-induced UC group; DSSPh, phloretintreated UC group (60 mg/kg/d). *n* = 6 for each group.

Overall, 471 metabolites and 11401 peaks were detected by LC-MS analysis from all samples. After normalization of the raw data, 9840 peaks were retained. The PCA (PC1: 36.9%; PC2: 11.6%) showed that the metabolic distributions of the three groups were separated, and the DSSPh group was closer to the Con group than to the DSS group (Fig. 2). Therefore, phloretin treatment improved the metabolic profiles of UC mice to some extent.

**Fig. 3:**
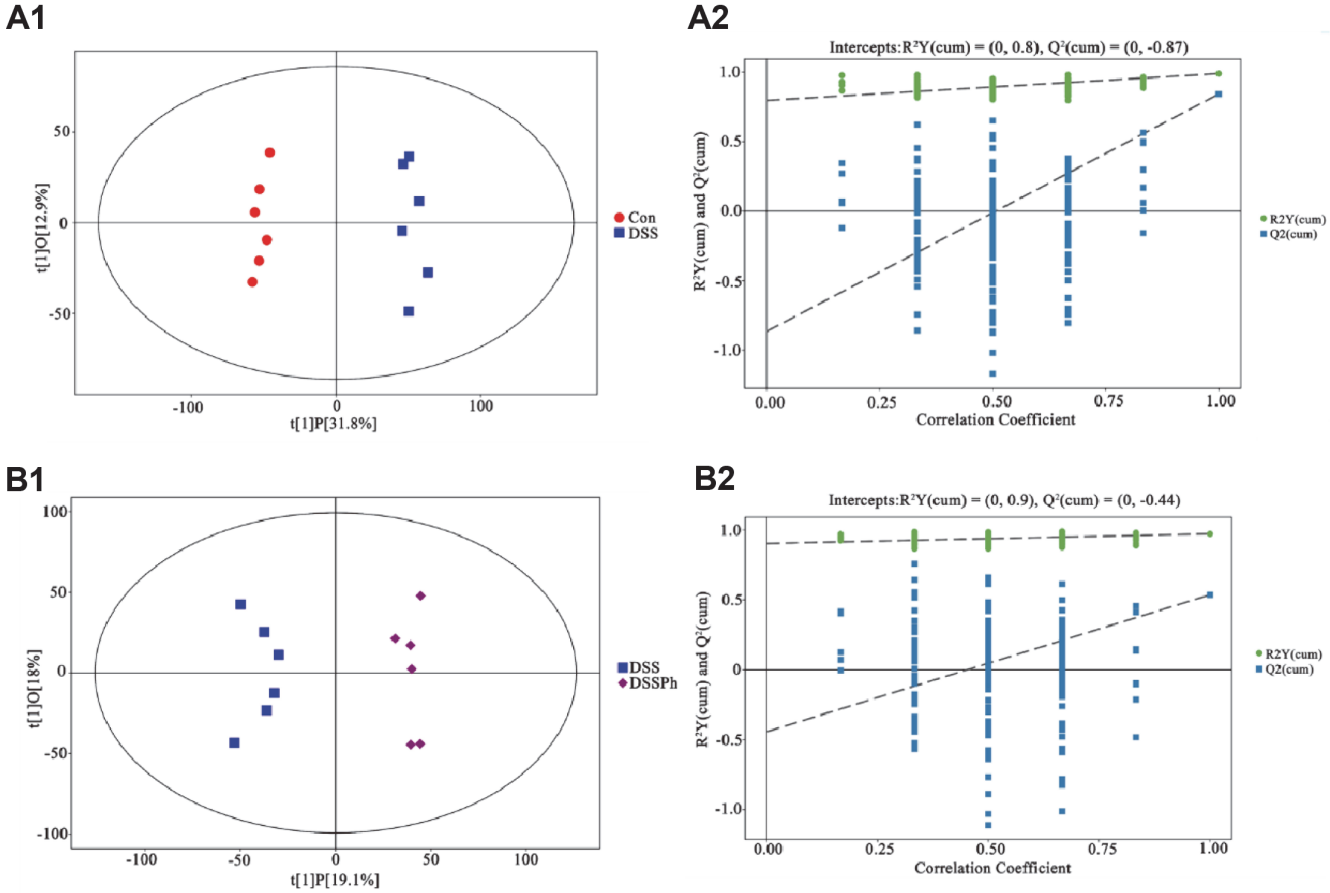
(A1) Score plot from OPLS-DA analysis of the metabolic profiles for the Con group vs. DSS group comparison; (A2): Permutation test of OPLS-DA for the Con group vs. DSS group comparison; (B1) Score plot from OPLS-DA analysis of the metabolic profiles for the DSS group vs. DSSPh group comparison; (B2): Permutation test of OPLS-DA for the DSS group vs. DSSPh group. Con, healthy control group; DSS: DSS-induced UC group; DSSPh, phloretin-treated UC group (60 mg/kg/d). *n* = 6 for each group.

To maximize class differences and better understand the variables responsible for the classification of the groups (Con group vs. DSS group; DSS group vs. DSSPh group), OPLS-DA was performed. As shown in Fig. 3, all samples in the score plot were in the 95% Hotelling T2 ellipse, and significantly different metabolic profiles were observed in these groups (Con group vs. DSS group; DSS group vs. DSSPh group). A 7-fold cross validation was conducted to explore the robustness and predictive ability of our model. After 200 permutations, the permutation test was used to further validate our model. The R^2^Y and Q^2^ intercept values of the Con group vs. DSS group comparison were 0.8 and -0.87, respectively. The R^2^Y and Q^2^ intercept values of the DSSPh group vs. DSS group comparison were 0.9 and -0.44, respectively. Therefore, the low intercept values of Q2 in these two comparisons indicated that our model was robust and was not overfitted.

### Compositions of Faecal Metabolites in the Different Groups

**Fig. 4:**
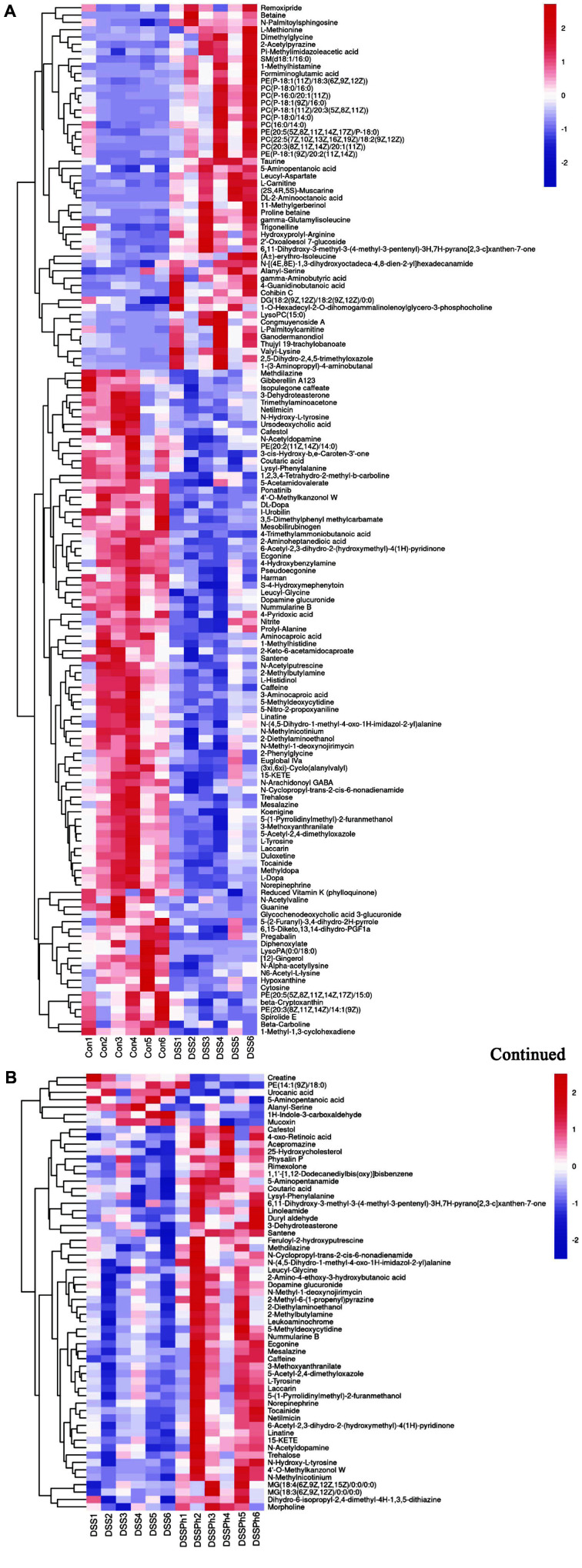
Heatmap of the results of hierarchical clustering analysis of the metabolite compositions. (**A**) Con group vs. DSS group; (**B**) DSS group vs. DSSPh group. The colour indicates the relative abundance of metabolites. Con, healthy control group; DSS: DSS-induced UC group; DSSPh, phloretin-treated UC group (60 mg/kg/d). *n* = 6 for each group.

OPLS-DA showed a loading plot of the contributions of different variables to the differences between the two groups. The loading plot was complex because it indicated many variables. The VIP value was used to improve this analysis and identify the characteristic metabolites among the three groups. A VIP value > 1 and a *p* value < 0.05 were the conditions used to select potential biomarkers. Overall, the levels of 50 metabolites were significantly higher in DSS group mice than in Con group mice, while the levels of 91 metabolites were significantly lower (Fig. 4A). Phloretin treatment significantly increased the levels of 52 metabolites and decreased those of 7 metabolites in the UC mice (Fig. 4B). The metabolites, listed in Table 2, had the highest MS2 scores (> 0.8), and their expression was significantly different among the Con, DSS and DSSPh groups. The changed metabolites were classified as phenylpropanoids and polyketides (4'-O-methylkanzonol W); organoheterocyclic compounds (N-methylnicotinium, laccarin, methdilazine, etc.); benzenoids (tocainide, mesalazine, norepinephrine, etc.); organic oxygen compounds (dopamine glucuronide and netilmicin), organic acids and derivatives (5-aminopentanoic acid, coutaric acid, lysyl-phenylalanine, etc.); lipids and lipid-like molecules (15-KETE, 3-dehydroteasterone, and N-cyclopropyl-trans-2-cis-6-nonadienamide); organic nitrogen compounds (*e.g.*, 2-diethylaminoethanol and 2-methylbutylamine); and nucleosides, nucleotides and analogues (5-methyldeoxycytidine). Their levels (except for 5-aminopentanoic acid and 6,11-dihydroxy-3-methyl-3-(4-methyl-3-pentenyl)-3H,7H-pyrano[2,3-c]xanthen-7-one) were lower in the DSS group than in the Con group. However, phloretin treatment reversed these effects, and the levels in the DSSPh group were significantly higher than those in the DSS group (Table 2). In addition, compared with the level in the Con group, only the level of 5-aminopentanoic acid was higher in the DSS group but lower in the DSSPh group. The level of 6,11-dihydroxy-3-methyl-3-(4-methyl-3-pentenyl)-3H,7H-pyrano[2,3-c]xanthen-7-one was higher in both the DSS group and DSSPh group than in the Con group, as shown in Table 2.

### Metabolic Pathway Analysis

**Fig. 5:**
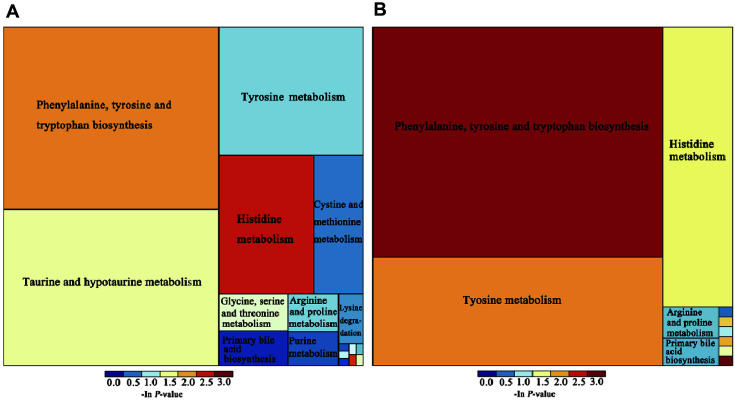
Treemap plot of the metabolic pathway. (**A**) Con group vs. DSS group; (**B**) DSS group vs. DSSPh group. The size of the square is positively related to the influence factor of the metabolic pathway. The legend shows the enrichment of the metabolic pathway. Con, healthy control group; DSS: DSS-induced UC group; DSSPh, phloretin-treated UC group (60 mg/kg/ d). *n* = 6 for each group.

The differential expression of metabolites in these groups reflected the changes in metabolic pathways. All of the metabolic pathways were connected and influenced by each other. In this research, we used the Kyoto Encyclopedia of Genes and Genomes (KEGG) database to research the metabolic pathways in which the different metabolites were joined. After filtering the metabolic pathways, we analysed the key metabolic pathways that had the highest correlations with differentially expressed metabolites. The treemap plot shows the differential metabolic pathways between groups (Con group vs. DSS group and DSS group vs. DSSPh group). As shown in Fig. 5, the metabolic pathways of “phenylalanine, tyrosine and tryptophan biosynthesis”, “tyrosine metabolism”, “histidine metabolism”, “arginine and proline metabolism”, and “primary bile acid biosynthesis” were significantly different in the DSS group than in the Con and the DSSPh groups. Thus, the results here indicate that phloretin treatment significantly improved metabolic pathways in UC mice.

### Correlation of the Gut Microbiota and Faecal Metabolites

**Fig. 6:**
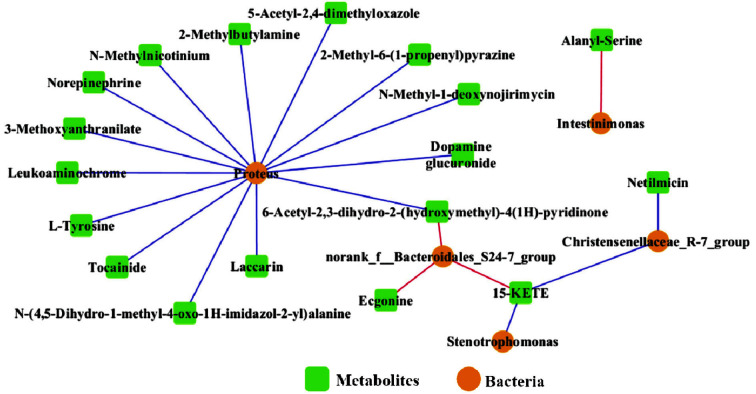
Network analysis of gut microbes and their metabolites. An edge indicates significant co-occurrence (red) or co-exclusion (blue). Con, healthy control group; DSS: DSS-induced UC group; DSSPh, phloretin-treated UC group (60 mg/kg/d). *n* = 6 for each group.

Spearman correlation coefficients based on heatmap analysis were used to research the relationships between the significantly changed gut microbes and their metabolites. The network diagram shows the microbes and metabolites with correlation coefficients > 0.8 and *p* < 0.5 (Fig. 6). The red line and blue line indicate positive and negative correlations between the gut microbes and their metabolites, respectively. As shown in Fig. 6, *Proteus* was negatively related to many faecal metabolites, such as norepinephrine, L-tyrosine, laccarin, dopamine glucuronide, and 5-acetyl-2,4-dimethyloxazole. The abundance of *norank_f__Bacteriodales_S24-7*_group was positively related to the levels of ecgonine, 15-KETE and 6-acetyl-2,3-dihydro-2-(hydroxymethyl)-4(1H)-pyridinone. The abundance of *Christensenellaceae*_R-7_group was negatively related to the levels of 15-KETE and netilmicin. *Stenotrophomonas* and 15-KETE were negatively related, while *Intestinimonas* and alanyl-serine were with positive related.

## Discussion

Phloretin is a dihydrochalcone flavonoid with antioxidant, anticancer and anti-inflammatory properties [28, 29]. Our previous study demonstrated the anti-UC effect of phloretin, which was regulated by the structure and composition of the gut microbiota [23]. It has been reported that substances extracted from dietary foods such as fruits and vegetables could be metabolized by gut microbes, and the metabolites from the gut microbes may then influence host health [30]. The phloretin treatment in our research was fed by gavage and then digested in the gastrointestinal tract; therefore, we reasoned that phloretin might also influence the faecal metabolite profile. As the effect of phloretin on the gut microbiota has been demonstrated in our previous research, in this research, we performed untargeted faecal metabolomics via LC-MS analysis and mainly focused on the changes in faecal metabolite profiles and the interactions between gut microbes and their metabolite profiles in phloretin-treated UC mice.

Increasingly, researchers have revealed that gut microbiota dysbiosis and faecal metabolic perturbations are associated with the development of diseases such as colitis-associated colon cancer, obesity, paediatric nonalcoholic fatty liver disease and Alzheimer’s disease [31-33]. In this study, gut microbes and their metabolites were both disturbed in UC mice. The results showed that phloretin treatment not only modulated the gut microbiota but also improved the faecal metabolite profile in UC disease mice, including by improving the metabolomic structure, composition and metabolic pathways. Moreover, a significant relation between the gut microbiota and faecal metabolites was observed. The network analysis indicated that *Proteus* had the highest correlations with metabolites, and all correlations were negative. Metabolites such as 2-methylbutylamine, N-methylnicotinium, 3-methoxyanthranilate, norepinephrine, tocainide, N-(4,5-dihydro-1-methyl-4-oxo-1H-imidazol-2-yl)alanine, laccarin, 6-acetyl-2,3-dihydro-2-(hydroxymethyl)-4(1H)-pyridinone, dopamine glucuronide, N-methyl-1-deoxynojirimycin and 5-acetyl-2,4-dimethyloxazole were negatively related to *Proteus*, and the abundances of these metabolites were all significantly lower in the DSS group than in the Con and DSSPh groups. Okumura *et al*. demonstrated that the presence of *Proteus* is correlated with colitis[34]. The abundance of *Proteus* in faeces and colon tissues is also significantly higher in *Lypd8^-/-^* mice, which are susceptible to DSS-induced colitis, than in WT mice[35].

We also observed that *Proteus* was negatively related to norepinephrine, and the abundance of norepinephrine in the DSS group was markedly lower than that in the Con and DSSPh groups. Recent studies have reported the role of norepinephrine in colitis. Mice with decreased concentration of norepinephrine in their colon tissues due to sympathectomy develop clinical symptoms of colitis and exhibit colon tissue oedema and increased colonic cytokines production [36]. The results in our study showed that the mRNA level of IL-10 was decreased in the DSS group but increased after phloretin treatment, and these changes were consistent with those of noradrenaline, the level of which was also decreased in the DSS group and increased in the DSSPh group. In addition, the levels of pro-inflammatory cytokines (IL-6 and IL-1β) were increased in the DSS group but decreased after phloretin treatment. Indeed, norepinephrine is involved in immune responses, and a previous study also reported that norepinephrine suppresses the secretion of pro-inflammatory cytokines such as IL-6, TNF-α, and IL-1β by inducing IL-10 secretion from innate cells [37]. Previous studies have focused mainly on the expression levels of norepinephrine in colon tissues; this study is the first to find a decreased abundance of norepinephrine in faeces from colitis mice, and to find that phloretin treatment significantly increased the abundance of norepinephrine. However, it remains to be further studied whether phloretin treatment directly affects the changes in *Proteus* and the levels of norepinephrine and whether the changes in cytokine levels are regulated by norepinephrine.

Moreover, phloretin treatment also significantly enhanced the abundance of mesalazine in UC mouse faeces. Mesalazine is the first-line medicine for treating UC patients [38]. The main ingredient of mesalazine is 5-aminosalicylate [39]. It has been recognized that 5-aminosalicylate inhibits prostaglandins and leukotrienes production and neutrophil chemotaxis and clears reactive oxygen metabolites [40]. As mesalazine was also detected in Con group mouse faeces, we reasoned that the existence of mesalazine might be due to the homeostasis of the gut microbiota. The improved gut microbial composition and structure may have resulted in production of the metabolite mesalazine. Although the effects of mesalazine on the composition of the gut microbiota have not been determined, Dahl reported that mesalazine directly alters the ability of microbes to colonize and survive within a chronic inflammatory environment [41]. The detailed role of mesalazine in the gut microbiota also needs further investigation. Besides, the abundance of 5-aminopentanoic acid was increased in the DSS group but decreased under phloretin treatment. A previous study has also reported that 5-aminopentanoic acid is associated with the later stages of intestinal inflammation [42]. However, in this study, no significant correlation was found between mesalazine or 5-aminopentanoic acid and other bacteria.

The changed abundance of metabolites impacted the metabolic pathways. KEGG pathway enrichment indicated that phloretin treatment was linked to changes in “phenylalanine, tyrosine and tryptophan biosynthesis”, “tyrosine metabolism”, “histidine metabolism”, “arginine and proline metabolism”, and “primary bile acid biosynthesis pathways”. The abundance of tyrosine was decreased in DSS group mice, while phloretin treatment significantly increased it. Tyrosine is the precursor of neurotransmitters and is beneficial for health under stress conditions [43]. Reduced expression of tyrosine has also been found to be related to a severe degree of inflammation in elderly people [44]. Moreover, changes in the gut microbiota in infants impact the tyrosine metabolism pathway [45]. In this study, the increased level of tyrosine might have been the result of phloretin acting on the gut microbiota in UC mice, which provides valuable information for further studies in the future.

Taken together, our findings demonstrated that phloretin improved the gut metabolism characteristics of mice with DSS-induced colitis, including their faecal metabolite profiles and metabolite compositions. The correlation analysis between the gut microbiota and faecal metabolites revealed that some changed faecal metabolites were closely correlated with the changed gut microbes. This result indicated that the improved gut microbiota after phloretin treatment was able to change the metabolic profile. In this study, only a few bacteria, especially *Proteus*, were found to be significantly correlated with metabolites, while some dominant bacteria, such as *Bacteroidetes*, *Alistipes* and *Lactobacillus*, were not found to be significantly correlated with metabolites; the study may have been limited by the untargeted metabolomics techniques. However, this study indicates the potential of the dietary ingredient phloretin to regulate faecal microbe metabolism for the treatment of UC and increases understanding of the detailed pharmacological mechanism of the protective effect of phloretin in mice with DSS-induced colitis.

## Supplemental Materials

Supplementary data for this paper are available on-line only at http://jmb.or.kr.

## Figures and Tables

**Table 1:** Primers used in the real-time PCR assays.

Gene	Primer Sequences (5’-3’)
TNF-α	Forward: CCCTCACACTCAGATCATCTTCT
	Reverse: GCTACGACGTGGGCTACAG
IL-6	Forward: TAGTCCTTCCTACCCCAATTTCC
	Reverse: TTGGTCCTTAGCCACTCCTTC
IL-1β	Forward: GCAACTGTTCCTGAACTCAACT
	Reverse: ATCTTTTGGGGTCCGTCAACT
IL-10	Forward: TGAGCAACTATTCCAAACCAGC
	Reverse: CGCAGCTCTAGGAGCATGTG
GAPDH	Forward: TCTGGAAAGCTGTGGCGTGAT
	Reverse: GCCAGTGAGCTTCCCGTTCAG

**Table 2:** Differentially expressed metabolites with VIP values > 1, *p* values < 0.05 and MS2 scores > 0.8 among the groups.

Metabolites	RT	Con	DSS	DSSPh	VIP	

					Con vs. DSS	DSS vs. DSSPh
Phenylpropanoids and polyketides						
4'-O-Methylkanzonol W	379.6945	0.152	0.026^***^	0.114^#^	1.629	1.979
Organoheterocyclic compounds						
N-Methylnicotinium	197.018	1.242	0.912^**^	1.572^###^	1.254	1.938
6-Acetyl-2,3-dihydro-2-(hydroxymethyl)-4(1H)-pyridinone	281.317	1.185	0.506^***^	0.765^##^	1.636	1.769
Laccarin	303.7555	1.294	0.702^***^	1.069^#^	1.500	1.187
Methdilazine	362.2655	0.912	0.392^**^	0.672^#^	1.166	1.518
5-Acetyl-2,4-dimethyloxazole	305.728	10.720	6.402^**^	9.273^#^	1.473	1.403
N-Methyl-1-deoxynojirimycin	392.469	1.016	0.691^**^	0.985^#^	1.237	1.405
6,11-Dihydroxy-3-methyl-3-(4-methyl-3-pentenyl)-3H,7H-pyrano[2,3-c]xanthen-7-one	364.945	0.066	0.200^*^	0.403^#^	1.235	1.666
Benzenoids						
Tocainide	243.444	0.565	0.292^***^	0.513^#^	1.485	1.490
Mesalazine	364.0465	2.530	0.937^**^	1.907^#^	1.481	1.523
3-Methoxyanthranilate	381.81	1.535	0.928^***^	1.338^#^	1.472	1.356
N-Acetyldopamine	268.421	0.409	0.307^**^	0.398^#^	1.278	1.463
Norepinephrine	322.407	5.170	2.418^***^	3.910^#^	1.601	1.369
Organic acids and derivatives						
5-Aminopentanoic acid	228.634	0.079	0.174^***^	0.125^#^	1.501	1.451
Coutaric acid	428.419	0.143	0.066^**^	0.137^##^	1.135	1.741
Lysyl-Phenylalanine	324.232	0.946	0.420^**^	0.795^#^	1.224	1.704
Linatine	370.251	2.018	1.255^*^	2.132^#^	1.178	1.719
Nummularine B	357.888	0.027	0.014^***^	0.025^##^	1.442	1.753
N-(4,5-Dihydro-1-methyl-4-oxo-1H-imidazol-2-yl)alanine	243.651	0.201	0.139^*^	0.203^#^	1.113	1.426
Lipids and lipid-like molecules						
15-KETE	256.287	0.272	0.163^**^	0.288^#^	1.311	1.533
N-Cyclopropyl-trans-2-cis-6-nonadienamide	194.588	0.279	0.212^**^	0.278^#^	1.377	1.616
3-Dehydroteasterone	54.9607	0.362	0.211^*^	0.324^#^	1.046	1.399
Organic nitrogen compounds						
2-Diethylaminoethanol	259.533	0.626	0.402^**^	0.663^#^	1.239	1.582
2-Methylbutylamine	253.125	1.858	1.000^**^	1.575^#^	1.370	1.443
5-(1-Pyrrolidinylmethyl)-2-furanmethanol	274.231	0.286	0.161^**^	0.243^#^	1.353	1.226
Nucleosides, nucleotides, and analogues						
5-Methyldeoxycytidine	103.326	7.167	3.979^**^	6.146^#^	1.369	1.488
Organic oxygen compounds						
Dopamine glucuronide	444.201	0.293	0.163^**^	0.255^##^	1.394	1.632
Netilmicin	82.084	0.981	0.510^**^	0.883^##^	1.456	1.750

*n* = 6 for each group. *p < 0.05, **p < 0.01, and ***p < 0.001 vs. Con group; ^#^*p* < 0.05, ^##^*p* < 0.01, and ^###^*p* < 0.001 vs. DSS group. RT: retention time. VIP: variable importance in the projection. MS2 score: the score of secondary matching is [0,1], and the higher the MS2 score is, the more reliable of the identification.
