# Supplementary material for: Interplay between the Gut Microbiome and Metabolism in Ulcerative Colitis Mice Treated with the Dietary Ingredient Phloretin
Source: J Microbiol Biotechnol. 2021 Aug 9;31(10):1409–19. doi: 10.4014/jmb.2104.04038 (PMC9705873; doi:10.4014/jmb.2104.04038)
Supplement: Supplementary file 1 [file jmb-31-10-1409-supple.pdf]

J. Microbiol. Biotechnol.
https://doi.org/10.4014/jmb.2104.04038

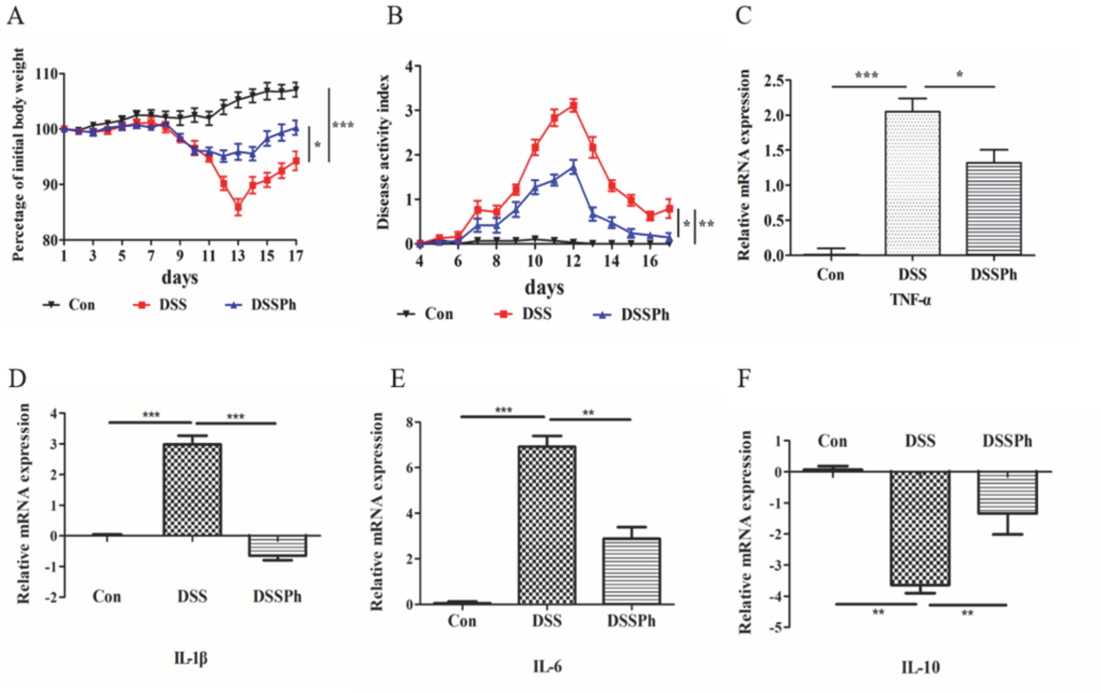

Figure S1. Ameliorative effect of phloretin on DSS-induced UC mice. (A) Body weight change. (B) DAI score during DSS treatment and the recovery period. (C-F) Relative mRNA levels of TNF-α, IL-6, IL-1β and IL-10 in colon tissues. Con, healthy control group; DSS: DSS-induced UC group; DSSPh, phloretin-treated UC group (60 mg/kg/d). These results are presented as the mean ± SEM; n = 6 for each group. $^{*}P < 0.05$, $^{**}P < 0.01$, $^{***}P < 0.001$.
